# The Impact of Wildfire Smoke on Acute Cardiovascular and Respiratory Illness in the US

**DOI:** 10.21203/rs.3.rs-9012485/v1

**Published:** 2026-04-09

**Authors:** Fintan Hughes, Luke Parsons, Brooke Alhanti, Jamarc Simon, Prasad Kasibhatla, Drew Shindell, Tetsu Ohnuma, Karthik Ragunathan, Hugh Montgomery, Vijay Krishnamoorthy

**Affiliations:** University College London; The Nature Conservancy; Duke University; Duke University; Duke University; Duke University; Duke University; Duke University; University College London; Duke University

**Keywords:** wildfire, particulate matter, cardiovascular health, respiratory health, public health

## Abstract

Escalating wildfire frequency increases population exposure to wildfire smoke. To evaluate the association between wildfire-specific particulate matter (PM_2.5_) and acute health impacts a retrospective cohort study was conducted utilizing National COVID Cohort Collaborative health records from 109,012 patients across 58 US health systems from 2020 to 2021. County-level wildfire-specific PM_2.5_ concentrations were estimated using a fire emissions database and chemical transport modeling. Generalized linear mixed-effects models were used to analyze the association between weekly county-level wildfire-specific PM_2.5_ exposure (up to 50 μg/m^3^) and hospital encounters for a series of cardiac, pulmonary, obstetric and neonatal outcomes. Statistically significant increases in weekly encounters per county of residence were observed for every 10 μg/m^3^ rise in weekly maximum wildfire-specific PM_2.5_ for acute myocardial infarction (0.084, 95% CI, 0.023–0.146), cardiac arrest (0.011, 95% CI, 0.001–0.021), heart failure (0.083, 95% CI, 0.024–0.142), atrial fibrillation (0.115, 95% CI, 0.014–0.216), COPD exacerbation (0.034, 95% CI, 0.001–0.066) and pulmonary embolism (0.048, 95% CI, 0.003–0.094). COVID infection status was not found to have a modifying effect on these relationships. There was no significant increase in COVID pneumonia admissions in response to increasing wildfire smoke. These findings demonstrate quantifiable increases in acute cardiorespiratory morbidity associated with wildfire smoke exposure.

## INTRODUCTION

Climate change, recognized as the greatest health threat of the 21st century ^[1]^, is driving an increase in wildfire frequency ^[2–4]^, and thus human exposure to wildfire smoke. From 2001–2004 to 2018–2021, 61% of countries experienced an increase in population exposure to wildfires ^[5]^. Inhalation of particulate matter (PM) from wildfire smoke triggers a cascade of harmful events, including inflammation, oxidative stress, endothelial dysfunction, sympathetic activation and platelet activation, which can result in end organ damage and lead to a variety of acute health issues ^[6]^. Direct contact of the respiratory system with PM increases the incidence of asthma and COPD exacerbation ^[7]^. Particulate matter with a diameter of 2.5 microns (PM_2.5_) and less is a particular focus in health research compared to larger particle sizes, as it penetrates deep into the lung and enters the circulation causing systemic effects ^[8]^. Systemic effects (above) can have a broad range of cardiovascular effects ^[9]^. Arrhythmias, including atrial fibrillation, are driven by inflammation and sympathetic stimulation ^[10]^. PM-induced hypertension increases cardiac afterload. Both can exacerbate heart failure ^[11]^. Platelet activation and endothelial dysfunction can precipitate acute coronary syndromes, myocardial infarction, ^[12]^ cerebral infarction and pulmonary embolism ^[13, 14]^. Ultimately, increased incidence of cardiac arrests has also been observed in response to PM exposure ^[15]^. Additionally, impacts on both the respiratory system and the endothelium are central to the pathophysiology of COVID-19 pneumonia, which was highly prevalent during the study period, and there appears to be a detrimental interaction between the effects of COVID-19 infection and PM exposure ^[16]^. The placental-fetal unit is also highly sensitive to endothelial dysfunction, inflammation and oxidative stress. Vascular injury and inflammation here can lead to pre-eclampsia, intrauterine growth restriction, premature labor and low birth weight ^[17, 18]^. Wildfire derived particulate matter appears to be more harmful than other sources of PM_2.5_, due to the presence of polycyclic aromatic hydrocarbons and aldehydes in the smoke ^[19]^.

Despite the increasing exposure of human populations to wildfire smoke, effective methods to predict and mitigate the health impact of this exposure remain inadequate. Although the mechanisms of smoke impact have been well described, little is known about the magnitude of the health impacts caused by exposure to given concentrations of wildfire smoke. Many existing studies have focused on the effects of individual wildfires in specific regions over brief time periods, or focused on the effects of isolated fire events on confined populations ^[20, 21]^. Many previous studies have tended to combine all PM sources, rather than isolating the effects of wildfire-specific PM_2.5_. Therefore, the differences between the health impacts of wildfire-specific (compared to anthropogenic) PM_2.5_ are not yet fully understood. A number of contemporary studies have examined the impact of wildfire-specific PM_2.5_, on a range of outcomes including cardiovascular and respiratory *mortality* globally ^[22]^, as well as in the US ^[23]^ and Brazil ^[24]^. However, impacts on *morbidity* (hospital admissions for specific diagnoses), and especially that relating to low-level exposure (such as that deriving from far distant fires) across large populations, are less well defined. While there are national level studies that estimate population-level respiratory impacts of PM exposure, they employ risk estimates from one or more smaller studies and extrapolate the risk estimates to a national level ^[25, 26]^. Our study improves on this methodology by measuring these outcomes directly. It is possible that significant unrecognized acute morbidity results from low-level wildfire smoke exposure. We sought to address this gap in knowledge, by studying the relationship between a range of cardiovascular, respiratory and obstetric/neonatal conditions and wildfire-related PM_2.5_ exposure (at the low-moderate concentrations, < 50 μg/m^3^, experienced by large populations) across the entire continental US (CONUS).

## RESULTS

**Figure 1:**
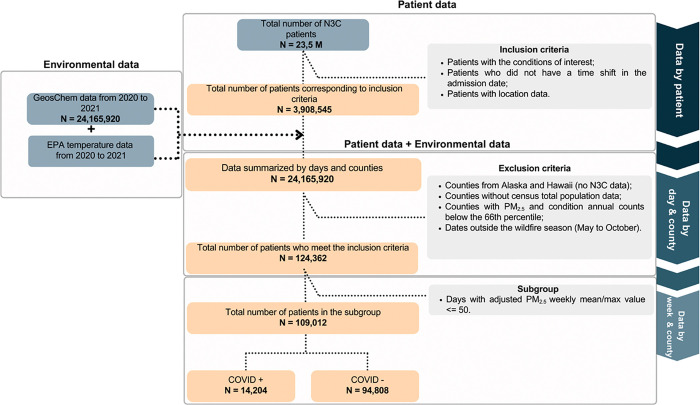
Flowchart illustrating data inclusion and exclusion criteria in the N3C study. Each of these values have been skewed by ±5 to enhance deidentification.

**Figure 2:**
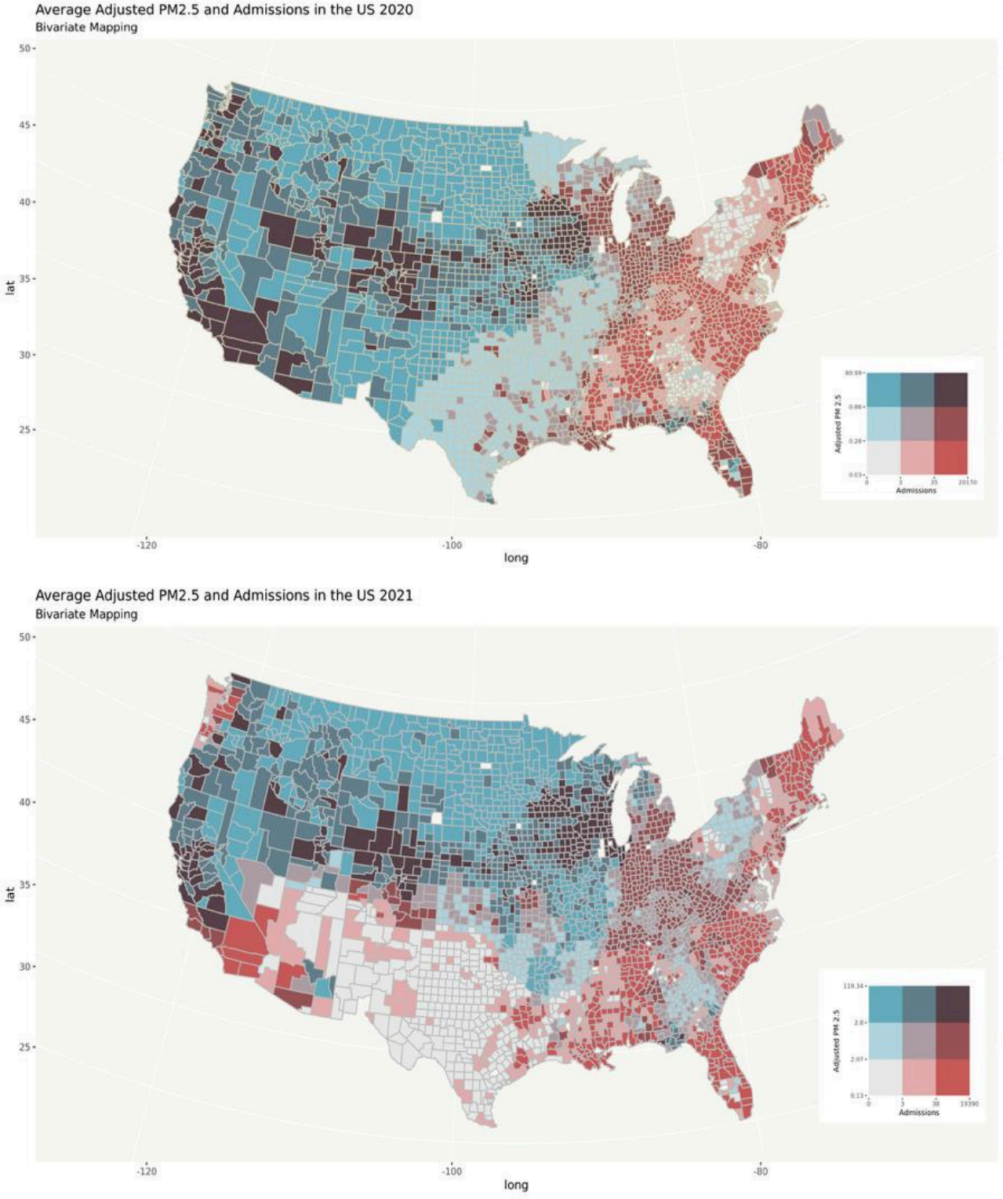
Bivariate maps of average adjusted PM_2.5_ concentration and hospital admissions in the United States (2020 above, 2021 below). The red gradient represents tertiles of mean weekly admission, and the blue gradient represents mean annual wildfire specific PM_2.5_. Only counties in the highest tertile of weekly admissions and wildfire specific PM_2.5_ were included. In 2020 this included counties with a mean annual wildfire specific PM_2.5_ concentration >0.86 μg/m^3^ and mean weekly admissions >35 patients. In 2021 included counties had a mean annual wildfire specific PM_2.5_ concentration >2.8 μg/m^3^ and mean weekly admissions >38 patients.

Figure 1 depicts a flowchart of the data selection process, utilizing the N3C database initially comprising a total of 23,155,003 patients. The analysis continued with restriction to 3,908,545 patients diagnosed with COVID-19, cardiac, pulmonary, obstetric, or neonatal conditions during the 2020–2021 period. Further exclusions were made for patients located outside CONUS, counties without available total population data, counties with annual admission rates and annual PM_2.5_ levels below the 66th percentile (Fig. 2), and dates outside of the wildfire season resulting in 124,362 patients. Finally, after removing days with county level wildfire-specific PM_2.5_ > 50 μg/m^3^, 109,012 patients remained in the dataset for analysis. Each of these values have been skewed by ± 5 to enhance deidentification.

Among patients treated at hospitals in the N3C database in areas affected by wildfire smoke, increasing wildfire-specific PM_2.5_ is associated with an increase in the incidence of major cardiovascular and respiratory diseases (Table 2). For each 10 μg/m^3^ increase in weekly maximum wildfire-specific PM_2.5_ there are statistically significant increases in the weekly incidence per county for acute myocardial infarction (0.084, 95% CI, 0.023–0.146), cardiac arrest (0.011, 95% CI, 0.001–0.021), heart failure (0.083, 95% CI, 0.024–0.142), atrial fibrillation (0.115, 95% CI, 0.014–0.216), COPD exacerbation (0.034, 95% CI, 0.001–0.066) and pulmonary embolism (0.048, 95% CI, 0.003–0.094). While the increased incidences of acute MI and heart failure exacerbations were consistent across all metrics of wildfire specific PM_2.5_ exposure, cerebral infarction only demonstrated an increased risk in relation to weekly mean wildfire specific PM_2.5_, and cardiac arrest correlated only with the weekly maximum concentration. There were no statistically significant increases in the incidence of obstetric or neonatal disease seen in this analysis. These results represent the mean increase in weekly hospital admissions and emergency department visits per county in response for each 10 μg/m^3^ increase in wildfire specific PM_2.5_.

No significant association was found between wildfire smoke exposure and hospital admission for COVID pneumonia or COVID-related respiratory distress. Similarly, our specificity analysis including 14,204 COVID positive patients did not demonstrate that COVID infection has a modifying effect on the impact of wildfire smoke exposure on hospital admissions for the measured acute medical admissions (Table 3).

The analyses were repeated, investigating the effects of total PM_2.5_, rather than wildfire specific PM on the same outcomes. In these analyses, very few of the same effects were observed as had been seen when studying the effects of wildfire PM_2.5_. When studying the effects of total PM exposure, acute myocardial infarction only showed an increase in response to the mean of the two weekly maximum values. Atrial fibrillation risk increased in response to both weekly maximum total PM and mean of the two weekly maximums. There was an increase in encounters for asthma in response to the mean of two weekly maximum values, which was not identified in wildfire specific analyses (Table 4).

## Discussion

Our findings suggest that each 10 μg/m^3^ rise in wildfire smoke exposure is associated with an increase in weekly county level hospital admission or emergency department presentation for acute myocardial infarction, cardiac arrest, heart failure, atrial fibrillation and pulmonary embolism. Weekly maximum PM_2.5_ concentrations most closely correlated with rates of admission. Such findings are significant as they demonstrate relatively low concentrations of wildfire smoke, in the range of 10–50 μg/m^3^, are associated with acute health impacts across a wide range of diagnoses impacting multiple organ systems.

We specifically sought to address the impact of lower-concentration and smoke-specific PM_2.5_ exposure and, as such, the magnitudes of the mean differences reported here are modest. However, our data suggest that beyond the immediate local effects of wildfires, large populations across a wide area may suffer associated acute health impacts, as smoke has been reported to travel hundreds of miles from large wildfires. As the population impacted by these low levels of smoke is large, so too are the associated economic impacts and burden to health systems. Importantly, the associations demonstrated in this work are specifically attributable to wildfire smoke at a national level, and are not limited to an individual fire, institution, or state. (Fig. 2) Hence, we propose that this approach makes these results more generalizable.

The lack of interaction between COVID infection and wildfire smoke may be explained by differences in the strength of each association, or a relatively small sample size of COVID positive patients. There is a shared mechanism of systemic inflammation and endothelial dysfunction between both COVID and wildfire smoke exposure. However, the acuity and severity of COVID infection requiring hospitalization may mask the effects of smoke exposure.

There are several notable contemporary studies that employ similar methodology to isolate wildfire specific PM using chemical atmospheric transport models. Applying a 14-day time series approach to national Brazilian mortality data demonstrated that wildfire specific PM exposure significantly increased risk of cardiovascular (2.6%) and respiratory (7.7%) mortality as well as all-cause mortality (2.4%). The outcome metric reported was the mean increase in mortality per 10 μg/m^3^ increase in wildfire specific PM_2.5_ over the two-week period. Significant geographic heterogeneity was observed in the results, with stronger relationships seen in the Southeast of Brazil, closer to the major population centers ^[24]^. A similar global time series, spanning 749 cities in 43 countries, was used to calculate an attributable fraction and relative risk of annual mortality from wildfire specific PM. For each 10 μg/m^3^ increase in wildfire specific PM_2.5_ across a moving 3 day average, relative risks were found to be 1.019, 1.017 and 1.019 for all-cause, cardiovascular and respiratory mortality, respectively ^[22]^. When studying the effect of smoke waves, defined as days with wildfire specific PM_2.5_ greater than 37 μg/m^3^, a 7.2% increase in all-cause respiratory admissions was observed among patients over the age of 65 in the Western United States. No significant increase was observed in cardiovascular admissions in response to these smoke waves ^[27]^. In Southern California, multiple derivations of wildfire-specific PM_2.5_ isolation and attributions demonstrate increased association with all-cause respiratory admissions compared to the association with total PM_2.5_. The effect of a 10 μg/m^3^ increase in daily ZIP code level wildfire specific PM_2.5_ ranged from 1.28% to 10% increase in respiratory admissions, depending on the approach used to isolate wildfire smoke ^[19]^. Our study is unique as it combines a large geographic area, long study period and diagnostic code level outcomes data with this contemporary approach to wildfire-specific PM_2.5_ isolation.

Our cohort was limited, as it did not capture every individual or admission within the regions studied. Due to the nature of the N3C dataset, only patients who were tested for COVID-19 were included. However, in the years 2020 and 2021, testing for COVID-19 became routine at many institutions, so it is unlikely that this induces significant selection bias. Moreover, the dataset includes data from only 58 hospital systems nationwide, meaning not all hospitals in the regions of interest were represented. As a result, the data are limited for California and the West Coast, areas of highest smoke exposure. Here, such sparse data create challenges, such as near-zero incidence rates when attempting to model the data at a daily temporal resolution despite use of zero-inflated models, which requiring a shift to weekly data. It is likely that the lack of daily analysis obscured the detection of respiratory presentations in this study. Furthermore, each hospital encounter is marked with the patient’s home ZIP code, which is not necessarily the area in which they experience smoke exposure. Additionally, as the exposure data were significantly skewed towards lower concentrations, the analysis was limited to concentrations of 50 μg/m^3^ and below. As a result of this narrower exposure range, a linear model was applied. While this limits our investigation of health impacts at higher smoke concentrations, it does make our model more representative of the exposure that much of the population likely experiences.

Future studies should aim to incorporate higher-fidelity health data with a more comprehensive spatial distribution. Capturing greater numbers of cases in regions of high wildfire smoke would allow for nonlinear modelling of the impacts of high concentration smoke exposure. Increased access to high quality health data will allow for improved modeling of the effects that have been seen in this study.

There is a significant increase in many cardiovascular and respiratory diseases in response to low concentration exposure to wildfire specific particulate matter. This observation at a population level demonstrates that even low concentrations of wildfire smoke, may cause acute health impacts.

## Methods

### Study Design

A retrospective cohort study was conducted to identify associations between exposure to wildfire-specific PM_2.5_ and hospital presentation with COVID-19 or acute cardiac and respiratory encounters, as well as two composite outcome measures of obstetric and neonatal pathology. This study was conducted using data from the National COVID Cohort Collaborative Secure Enclave, which were collected under Johns Hopkins University School of Medicine Central IRB (IRB00249128). Based on 45 CRF 46.116 guidelines, a waiver of informed consent was granted by the John Hopkins University IRB. The Institutional Review Board of Duke University determined that our study was exempt as the work deals exclusively with deidentified data (Pro00109375). All methods were carried out in accordance with relevant guidelines and regulations. All experimental protocols were approved by the above named institutional committees.

### Database

Electronic health record data during US wildfire season (May 1st to October 31st) from the National COVID Cohort Collaborative (N3C) for 2020 and 2021 were evaluated. N3C is an extensive centralized repository of all patients who underwent COVID-19 testing in 58 different healthcare systems across the United States ^[28]^. This work took place under an N3C Data Use Request [DUR-5477342]. Importantly, COVID testing was performed routinely in these hospital systems during the study period. This includes patients who presented to emergency departments or were admitted inpatient at any of the hospitals that report to N3C. The N3C limited dataset uses diagnostic codes in the form of Observational Medical Outcomes Partnership Common Data Model (OMOP-CDM). The database contains daily counts of encounters, labelled with OMOP-CDM diagnostic codes and associated ZIP codes for the patients’ home addresses. Our function selects the first relevant diagnostic code to determine the cause of the encounter. To preserve temporal accuracy, we filtered the dataset to include only records without date shifting, which is a practice employed in some instances to deidentify records.

### Population

Inclusion criteria: All Adult (≥ 18 years) and neonatal (< 1 month) patients in the N3C database, where the visit was coded with one or more of the diagnoses specified for investigation (Supplemental Matieral 1).

Exclusion criteria: Encounters that lacked localization or visit information data, or patients outside the continental US (Hawaii and Alaska states) were excluded from the study. Additionally, days falling outside the wildfire season (above) and outlier days with county-level wildfire-specific PM_2.5_ concentrations > 50 μg/m^3^ were excluded. A threshold of 50 μg/m^3^ was chosen, as above this concentration our models became unreliable due to sparse data.

To avoid modelling areas with minimal smoke exposure, patients residing in counties falling in the bottom 66% for annual mean wildfire PM_2.5_ concentrations and annual mean admissions each year were excluded. This was required to overcome problems of near-zero incidence, in counties with sparse population and few admissions, and to exclude areas with minimal smoke exposure throughout the study period.

### Data Processing

Patient outcomes were aggregated into counts per day by patient home ZIP Code. The outcome data were then merged with the PM_2.5_ exposure data from CAMS and GeosCHEM (below) using the day and ZIP Code as standard identifiers. Finally, to overcome modelling limitations in cases of near zero incidence when using daily admission data, the data were aggregated at the county level and compiled into weekly counts. The cohort construction process is illustrated in the flowchart in Fig. 1.

### Exposure

Daily total PM_2.5_ in micrograms per cubic meter (μg/m^3^) was evaluated using data from the Copernicus atmosphere monitoring service (CAMS) ^[29]^. To isolate the component of total PM concentrations that is attributable to wildfire smoke, a chemical transport model was employed. Specifically, the GEOS-Chem atmospheric model ^[30]^ was run both with and without satellite fire emissions data from NASA’s Global Fire Emissions Database v4. Initially, a global model was run at 4° × 5° spatial resolution. With these boundary conditions, 0.5° × 0.625° models were run for the continental US. From this modelling, a ‘fire fraction’ was calculated at each geographical and temporal data point to represent the proportion of total PM_2.5_ that was specifically attributable to wildfires. The fire-fraction data from GEOS-Chem were then used to apportion the original CAMS data, with the output taken to represent wildfire specific PM_2.5_ concentrations, as previously described ^[27]^. Values for wildfire-specific PM_2.5_ were calculated from 2020 through 2021 for each CONUS ZIP Code and were then averaged at the US county level. Three separate exposure metrics were used in our analysis. First, the mean of the two maximum weekly wildfire-specific PM_2.5_ values was investigated with a one-day lag. This metric, similar to that used in other studies, captures periods of ongoing smoke exposure while accounting for the delay between exposure and disease onset or hospitalization ^[31]^. Addition exposure variables were of weekly maximum and weekly mean wildfire-specific PM_2.5_ concentrations.

### Outcomes

The outcomes examined were the weekly incidence of acute myocardial infarction, acute heart failure exacerbation, atrial fibrillation, cerebral infarction, pulmonary embolism, cardiac arrest, asthma, and acute exacerbation of COPD at a county level. To investigate the sensitivity of the fetal-placental vasculature to wildfire smoke exposure, composite measures for adverse neonatal and obstetric outcomes were also included. The complete list of OMOP-CDM concepts associated with these categories is presented in Supplemental Matieral 1.

### Covariates

Covariates included the month of the year, the total (non-wildfire-specific) PM_2.5_ exposure, county level COVID-19 incidence, and temperature difference from monthly means.

### Statistical Methods

Generalized linear mixed-effects models, adjusting for previously identified covariates, were used. The results are expressed as fixed-effect coefficients describing the change in weekly county level incidence of each condition with each 10 μg/m^3^ increase in wildfire specific PM_2.5_, concentrations, along with 95% confidence intervals. Results were considered statistically significant when p-values were below 0.05 and when the confidence intervals did not include zero. The analyses were then repeated, investigating the effects of total PM_2.5_, rather than wildfire specific PM on the same outcomes.

### COVID interaction

A separate generalized linear mixed effects model was run to investigate the impact of wildfire smoke on COVID-positive admissions, defined as those with a COVID diagnosis within ± 7 days of admission. This model did not include COVID incidence as a confounder and instead measured the rate of admission for COVID associated pneumonia, respiratory distress or hypoxia. Additionally, a sensitivity analysis was performed to investigate differences in the association between wildfire smoke and admission rates among patients with and without comorbid COVID infection. All analyses were conducted using the R programming language, including figures, which were created with the R ggplot 2, and biscale packages ^[32]^.

## Supplementary Material

This is a list of supplementary files associated with this preprint. Click to download.

• SupplementalMaterial.docx

## Figures and Tables

**Table 1:** Characteristics of the study sample stratified by year (2020–2021)

n	Overall	2020	2021
	109,012¥	54,272¥	54,740¥
Length of stay (mean (SD))	2.31 (8.44)	2.29 (8.26)	2.34 (8.63)
Gender (%)			
- Male	51,694 (47.4)	25,828 (47.6)	25,866 (47.3)
- Female	57,299 (52.6)	28,435 (52.4)	28,864 (52.7)
- Other/Unknown	< 20* (0.0)	< 20* (0.0)	< 20* (0.0)
Race (%)			
- White	70,397 (64.6)	35,344 (65.1)	35,053 (64.0)
- Black or African American	17,136 (15.7)	8,266 (15.2)	8,870 (16.2)
- Asian	4,297 (3.9)	2,053 (3.8)	2,244 (4.1)
- Other/Unknown	17,182 (15.8)	8,609 (15.9)	8,573 (15.7)
Age (mean (SD))	51.53 (25.11)	52.44 (23.82)	50.63 (26.30)
Death (%)	13,226 (12.1)	7,401 (13.6)	5,825 (10.6)
Quan-Charlson comorbidity score (mean (SD))	1.53 (2.00)	1.51 (1.97)	1.56 (2.03)
Covid status = Positive (%)	14,204 (13.0)	6,745 (12.4)	7,459 (13.6)
Estimated percent poverty (mean (SD))	12.57 (4.21)	12.57 (4.22)	12.56 (4.21)
Estimated percent unemployment (mean (SD))	5.37 (1.84)	5.38 (1.85)	5.35 (1.83)
Estimated per capita income (mean (SD))	34,590.71 (7,385.32)	34,506.52 (7,217.05)	34,674.19 (7,547.59)
Estimated percent no high school diploma (mean (SD))	10.74 (4.64)	10.74 (4.54)	10.73 (4.74)
Acute myocardial infarction (%)	11,744 (10.8)	5,946 (11.0)	5,798 (10.6)
Atrial fibrillation (%)	22,534 (20.7)	11,721 (21.6)	10,813 (19.8)
Cardiac arrest (%)	2,306 (2.1)	1,142 (2.1)	1,164 (2.1)
Heart failure (%)	8,319 (7.6)	4,334 (8.0)	3,985 (7.3)
Chronic obstructive pulmonary disease (%)	4,647 (4.3)	2,186 (4.0)	2,461 (4.5)
Asthma (%)	26,379 (24.2)	12,499 (23.0)	13,880 (25.4)
Pulmonary embolism (%)	7,521 (6.9)	3,673 (6.8)	3,848 (7.0)
Cerebral infarction (%)	10,626 (9.7)	5,364 (9.9)	5,262 (9.6)
Neonatal cases (%)	5,498 (5.0)	2,839 (5.2)	2,659 (4.9)
Obstetric cases (%)	7,960 (7.3)	4,215 (7.8)	3,745 (6.8)

* Counts less than 20 were masked for data privacy purposes.

¥ Values were skewed by up to 5 to obscure precise counts.

**Table 2:** Results of generalized linear mixed effects model, with three different exposure metrics for weekly particulate matter exposure as predictive variables. Columns show the weekly county level change in incidence per 10 μg/m^3^ increase in **wildfire-specific** PM_2.5_. Confounders include month, the difference in weekly temperature from monthly mean, total PM_2.5_, and county level COVID-19 incidence.

Outcome	Mean of maximum 2 values of PM2.5			Weekly maximum PM2.5			Weekly mean PM2.5		
	Change in incidence per county per week/10 μg/m^3^	CI	P value	Change in incidence per county per week/10 μg/m^3^	CI	P value	Change in incidence per county per week/10 μg/m^3^	CI	P value
Acute myocardial infarction	**0.095**	**[0.012–0.177]**	**0.025**	**0.084**	**[0.023–0.146]**	**0.007**	**0.134**	**[0.017–0.251]**	**0.025**
Cardiac arrest	0.011	[−0.003–0.024]	0.13	**0.011**	**[0.001–0.021]**	**0.036**	0.018	[−0.002–0.038]	0.074
Heart failure	**0.095**	**[0.015–0.174]**	**0.019**	**0.083**	**[0.024–0.142]**	**0.006**	**0.135**	**[0.022–0.248]**	**0.019**
Atrial fibrillation	0.111	[−0.023–0.246]	0.104	**0.115**	**[0.014–0.216]**	**0.026**	0.186	[−0.005–0.378]	0.057
Cerebral infarction	0.068	[−0.009–0.145]	0.082	0.043	[−0.015–0.100]	0.147	**0.189**	**[0.079–0.298]**	**<0.001**
COPD	**0.05**	**[0.007–0.093]**	**0.024**	**0.034**	**[0.001–0.066]**	**0.041**	0.049	[−0.013–0.111]	0.122
Asthma	0.036	[−0.356–0.429]	0.857	0.067	[−0.229–0.362]	0.659	0.054	[−0.492–0.601]	0.845
Pulmonary embolism	**0.077**	**[0.016–0.139]**	**0.014**	**0.048**	**[0.003–0.094]**	**0.038**	0.076	[−0.013–0.164]	0.095
*COVID hospitalization	0.045	[−0.268–0.359]	0.776	0.026	[−0.147 – 0.2]	0.767	0.742	[−0.046–1.529]	0.065
Neonatal outcomes	0.027	[−0.031–0.085]	0.356	0.03	[−0.014–0.073]	0.179	0.059	[−0.023–0.14]	0.157
Obstetric outcomes	0.023	[−0.052–0.099]	0.545	0.038	[−0.018–0.094]	0.459	0.04	[−0.068–0.148]	0.468

* When measuring the rate of COVID admissions, a separate model was run that did not include county level COVID-19 incidence as a confounding variable.

**Table 3:** Specificity analysis comparing COVID positive and COVID negative patients, using weekly maximum wildfire specific PM_2.5_ as the predictive variable. Columns show the weekly county level change in incidence per 10 μg/m^3^ increase in **wildfire-specific** PM_2.5_. Confounders include month, the difference in weekly temperature from monthly mean, and total PM_2.5_.

Outcome	COVID +			COVID −		
	Change in incidence per county per week/10 μg/m^3^	CI	P value	Change in incidence per county per week/10 μg/m^3^	CI	P Value
Acute myocardial infarction	0.016	[−0.036–0.068]	p = 0.553	0.092	[−0.066–0.251]	p = 0.252
Cardiac arrest	0.001	[−0.023–0.025]	p = 0.942	−0.031	[−0.097–0.036]	p = 0.367
Heart failure	−0.016	[−0.054–0.022]	p = 0.416	−0.009	[−0.137–0.119]	p = 0.885
Atrial fibrillation	0.056	[−0.020–0.131]	p = 0.148	−0.115	[−0.302–0.071]	p = 0.226
Cerebral infarction	−0.013	[−0.053–0.026]	p = 0.504	−0.104	[−0.241–0.033]	p = 0.136
COPD	0.044	[−0.014–0.103]	p = 0.136	0.063	[−0.038–0.164]	p = 0.222
Asthma	−0.002	[−0.077–0.072]	p = 0.948	0.018	[−0.142–0.178]	p = 0.827
Pulmonary embolism	0.032	[−0.026–0.090]	p = 0.281	−0.035	[−0.138–0.068]	p = 0.506
Neonatal outcomes	−0.011	[−0.039–0.016]	p = 0.422	0.021	[−0.040–0.082]	p = 0.498
Obstetric outcomes	−0.001	[−0.026–0.025]	p = 0.958	−0.049	[−0.165–0.067]	p = 0.408

**Table 4:** Results of generalized linear mixed effects model, with three different exposure metrics for weekly particulate matter exposure as predictive variables. Columns show the weekly county level change in incidence per 10 μg/m^3^ increase in **total** PM_2.5_. Confounders include month, the difference in weekly temperature from monthly mean, total PM_2.5_, andcounty level COVID-19 incidence.

Outcome	Mean of maximum 2 values of PM_2.5_			Weekly maximum PM_2.5_			Weekly mean PM_2.5_		
	Change in incidence per county per week/10 μg/m^3^	CI	P value	Change in incidence per county per week/10 μg/m^3^	CI	P value	Change in incidence per county per week/10 μg/m^3^	CI	P value
Acute myocardial infarction	0.181	[0.001–0.361]	0.049	0.085	[−0.117–0.287]	0.41	0.402	[−0.106–0.911]	0.121
Cardiac arrest	0.018	[−0.051–0.087]	0.612	0.048	[−0.030–0.125]	0.232	−0.016	[−0.212–0.180]	0.871
Heart failure	0.103	1 [−0.051–0.087]	0.151	−0.019	[−0.177–0.138]	0.809	0.131	[−0.266–0.528]	0.517
Atrial fibrillation	**0.355**	**[0.100–0.611]**	**0.006**	0.222	[−0.065–0.509]	0.13	0.89	[0.168–1.611]	0.016
Cerebral infarction	0.111	[−0.056–0.278]	0.193	0.069	[−0.119–0.256]	0.474	0.408	[−0.063–0.880]	0.09
COPD	0.055	[0.100–0.611]	0.4	0.017	[−0.128–0.161]	0.822	0.153	[−0.210–0.516]	0.408
Asthma	**0.302**	**[0.064–0.540]**	**0.013**	0.188	[−0.079–0.455]	0.168	0.577	[−0.095–1.249]	0.093
Pulmonary embolism	−0.049	[−0.188–0.090]	0.487	−0.015	[−0.171–0.141]	0.853	−0.248	[−0.641–0.144]	0.215
Neonatal outcomes	0.014	[−0.100–0.127]	0.814	−0.032	[−0.159–0.096]	0.628	0.104	[−0.216–0.424]	0.524
Obstetric outcomes	0.063	[−0.074–0.199]	0.368	0.112	[−0.042–0.265]	0.153	0.2	[−0.185–0.585]	0.308

## Data Availability

The health outcomes data used in this study are available through the National COVID Cohort Collaborative (N3C) Secure Enclave, maintained by NCATS. Researchers may access the data by submitting a Data Use Request (DUR) via the N3C portal (covid.cd2h.org).Atmospheric data supporting the exposure metrics are publicly available. Total PM2.5 data were obtained from the Copernicus Atmosphere Monitoring Service (CAMS) (https://ads.atmosphere.copernicus.eu/datasets/cams-global-reanalysis-eac4). Wildfire emission data were sourced from the NASA Global Fire Emissions Database (GFEDv4) (https://globalfiredata.org/related.html#gfed4). The GEOS-Chem model code used for chemical transport modeling is open-source and available at geos-chem.org. Processed wildfire-specific PM2.5fractions generated during this study are available from the corresponding author upon request
